# Prevalence of multimorbidity in adults with cancer, and associated health service utilization in Ontario, Canada: a population-based retrospective cohort study

**DOI:** 10.1186/s12885-021-08102-1

**Published:** 2021-04-14

**Authors:** Anna Péfoyo Koné, Deborah Scharf

**Affiliations:** 1 Department of Health Sciences, Lakehead University, 955 Oliver Rd, Thunder Bay, ON P7B 5E1 Canada; 2 Department of Psychology, Lakehead University, 955 Oliver Rd, Thunder Bay, ON P7B 5E1 Canada

**Keywords:** Cancer, Multimorbidity, Chronic disease, Health service utilization, Mortality

## Abstract

**Background:**

The majority of people with cancer have at least one other chronic health condition. With each additional chronic disease, the complexity of their care increases, as does the potential for negative outcomes including premature death. In this paper, we describe cancer patients’ clinical complexity (i.e., multimorbidity; MMB) in order to inform strategic efforts to improve care and outcomes for people with cancer of all types and commonly occurring chronic diseases.

**Methods:**

We conducted a population-based, retrospective cohort study of adults diagnosed with cancer between 2003 and 2013 (*N* = 601,331) identified in Ontario, Canada healthcare administrative data. During a five to 15-year follow-up period (through March 2018), we identified up to 16 co-occurring conditions and patient outcomes for the cohort, including health service utilization and death.

**Results:**

MMB was extremely common, affecting more than 91% of people with cancer. Nearly one quarter (23%) of the population had five or more co-occurring conditions. While we saw no differences in MMB between sexes, MMB prevalence and level increased with age. MMB prevalence and type of co-occurring conditions also varied by cancer type. Overall, MMB was associated with higher rates of health service utilization and mortality, regardless of other patient characteristics, and specific conditions differentially impacted these rates.

**Conclusions:**

People with cancer are likely to have at least one other chronic medical condition and the presence of MMB negatively affects health service utilization and risk of premature death. These findings can help motivate and inform health system advances to improve care quality and outcomes for people with cancer and MMB.

**Supplementary Information:**

The online version contains supplementary material available at 10.1186/s12885-021-08102-1.

## Introduction

Multimorbidity (MMB), defined as the co-occurrence of multiple chronic conditions, is a public health crisis, challenging healthcare systems and affected individuals and their families [1–5]. The risk of MMB is particularly high among people with cancer [4, 6–10]. In Ontario, Canada, more than 75% of people with cancer have at least one of 16 other prevalent chronic conditions [4]. Rates of MMB among cancer patients, however, vary by cancer type [6, 11, 12]. Similarly, the chronic condition(s) that co-occur with cancer also vary by cancer type and can have different impacts on patient outcomes, including death [11, 13, 14].

MMB impacts people with cancer directly through an increased physiological burden of disease and/or indirectly through healthcare factors related to treatment decision-making and the increased complexity of care [15]. For example, MMB can complicate and delay cancer and other diagnoses, increase treatment complexity, risk of polypharmacy, and/or create other redundancies in care [16–20]. Cancer patients with MMB are less likely to be offered active or curative therapies [21–23], despite growing evidence that such treatments can be tolerated and effective for some patients [24–26]. People with cancer and MMB likely also experience increased burden from managing multiple appointments and care regimens, which may result in confusion, stress, difficulty adhering to treatment protocols, and poor quality of life.

Another critical challenge to effectively treating and improving outcomes for people with cancer and other chronic conditions is that most care plans follow a single disease-oriented approach [10, 18, 27, 28]. In such single-disease models, the management of co-occurring conditions often takes a backseat to cancer even when coordinated management of chronic conditions could help improve patient outcomes and quality of life [29, 30]. Well-documented gaps in clinical guidelines and care management frameworks demonstrate the need for intentional integration and coordination of cancer and chronic disease care, including clear direction to providers who may lack clarity about their roles and responsibilities [31–33] for patients receiving services spanning siloed specialized and primary care settings [2, 34–36].

In order to improve care and outcomes for people with cancer and MMB, health care systems must recognize and reorganize care delivery in order to provide whole-person care. However, few large epidemiological studies have examined the overall extent of MMB among people with cancer of all types to provide guidance about how care should be improved. Similarly, researchers have not broadly documented the potential impact of cancer MMB on health service utilization at the general population level in order to motivate care delivery reform.

In this paper, we address these research gaps by describing the co-occurrence of cancer of any type with 16 common chronic diseases, as well as the level of MMB associated with patients’ health service utilization in an Ontario, Canada population-based, retrospective cohort study. In a second, companion paper using this same data set, we examine which conditions are most likely to co-occur with cancer and how specific disease clusters impact patient outcomes (Koné et al., under review).

## Methods

### Study design and data sources

We performed a retrospective cohort study of people diagnosed with cancer in Ontario, Canada using population-based healthcare administrative data. The province of Ontario provides universal coverage of physician visits and hospital services for virtually all residents. This study population includes all individuals 18 years and older living in Ontario with a valid health card, who received a cancer diagnosis between April 1, 2003 and March 31, 2013. Individuals were then followed-up through March 31, 2018 in order to track the occurrence of 16 co-occurring chronic health conditions and health service utilization (HSU).

As in our previous work [4], data came from the Institute for Clinical Evaluative Sciences (ICES) linked data sets including information from: The Registered Persons Database (RPDB), the Canadian Institute for Health Information’s Discharge Abstract Database (DAD), the Ontario Health Insurance Plan (OHIP) claims database, the Ontario Drug Benefits (ODB), and the National Ambulatory Care Reporting System (NACRS). ICES used unique encoded identifiers to create the study population, to identify occurrence of conditions, and to measure health service utilization. ICES made the cohort available on the secure (IDAVE) website for remote analyses by the researchers. The Lakehead University Research Ethics Board reviewed and approved this research.

### Measures

#### Multimorbidity

We defined MMB as the co-occurrence of any of 16 conditions specified below with any cancer diagnosis, similar to previous research [4, 37, 38].

#### Multimorbidity level

We defined and grouped individuals by MMB level as defined by the presence of one, two, three, four, or five or more conditions, as compared to cancer only.

#### Co-occurring conditions

We identified the presence of 16 chronic conditions using hospital discharge (DAD), physician billing (OHIP) and drug prescription (ODB) data. These conditions included: acute myocardial infarction (AMI), asthma, cancer, cardiac arrhythmia, chronic obstructive pulmonary disease (COPD), congestive heart failure (CHF), chronic coronary syndrome, dementia, diabetes, hypertension (HT), non-psychotic mood and anxiety disorders, other mental illnesses, osteoarthritis, osteoporosis, renal failure, rheumatoid arthritis, and stroke.

#### Cancer

We used cancer ICD-O-3 topography and histology codes and stage indicators available from the Ontario Cancer Registry (OCR). Cancer type was then defined according to SEER program [39] for site groups.

#### Health services utilization (HSU)

We included several measures of HSU including number of primary care (PC) visits, emergency department (ED) visits, and hospital admissions. To account for different lengths of follow-up and/or death, we defined HSU in two ways for individuals with more than 30 days of follow-up post cancer diagnosis: 1) by counts during the first-year post cancer diagnosis, and 2) average person-year counts for entire follow-up.

#### Survival and mortality

Survival was defined as time to all-cause death during follow-up, using death date from the RPDB. Those without the event were censored at maximum follow-up time (i.e., systematic treatment of individuals for whom incomplete information is available), as this is standard practice for survival analyses [40]. Rate of mortality was also calculated within 1 year, 5 years, and overall during follow-up for those who survived at least 30 days post cancer diagnosis. Mortality was the outcome of interest for the multivariate regression.

#### Demographics

Basic demographic variables including age, sex, and geographic region of residence were obtained from the RPDB.

### Analyses

We used descriptive statistics to characterize population demographics, common cancer sites, and MMB level. We then used bivariate and age-stratified analyses to assess cancer complexity relative to participant demographics, cancer type, HSU, and mortality. Survival curves stratified by multimorbidity levels and cancer stage at diagnosis, were created using Kaplan-Meier method. Finally, we used negative binomial and logistic regressions to assess the adjusted impact of increasing cancer complexity on health service utilization and mortality (risk of death) within the first year, using pre-existing MMB levels (i.e. up to 30 days after cancer diagnosis) and controlling for age, sex, cancer types, and cancer stage at diagnosis. We used SAS v.10 to conduct all analyses.

## Results

Our study population included 601,331 adults diagnosed with cancer between 2003 and 2013; 49% were females and 55.7% aged 65 years or older. The most common types of cancer identified in the sample were (in descending order): Prostate, breast, colon/rectum, and lung/bronchus (Table 1).

**Table 1:** Population characteristics: Baseline and follow-up (*N* = 601,331)

Characteristics	N (%)
**Female Sex**	294,490 (49)
**Age group**	
18–44	49,362 (8.2)
45–64	217,229 (36.1)
65+	334,740 (55.7)
**Rural residency**	85,547 (14.2)
**Region**	
Central	190,015 (31.6)
Central east	94,127 (15.7)
East	83,660 (13.9)
North	45,937 (7.6)
South	104,718 (17.4)
Southwest	82,592 (13.7)
**Cancer type**	
Brain and Other Nervous System	8553 (1.4)
Breast	81,553 (13.6)
Cervix Uteri	5197 (0.9)
Colon and Rectum	71,754 (11.9)
Digestive System, except Colon	49,621 (8.3)
Endocrine System	22,564 (3.8)
Female Genital System, except	30,983 (5.2)
Leukemia	16,996 (2.8)
Lung and Bronchus	75,619 (12.6)
Lymphoma	28,437 (4.7)
Myeloma	8487 (1.4)
Oral Cavity and Pharynx	13,715 (2.3)
Other	39,691 (6.6)
Prostate	87,377 (14.5)
Skin excluding Basal and Squam	23,952 (4)
Urinary System	36,832 (6.1)
**Stage of cancer**	
1	80,182 (13.3)
2	103,148 (17.2)
3	61,109 (10.2)
4	69,693 (11.6)
unknown	287,199 (47.8)
**Multimorbidity**	
No additional condition	52,083 (8.7)
1	97,478 (16.2)
2	118,310 (19.7)
3	109,166 (18.2)
4	85,077 (14.1)
5 or more conditions	139,217 (23.2)
**Follow-up in years:** Average (SD); range	5.8 (4.5); 0^a^ - 15
**Health service use within the first year (follow-up > =30 days**^**b**^**)**	
**Primary care visit**: at least one	519,752 (92.2)
Primary care visit: High use (90th pct, i.e. > = 15)	63,835 (11.4)
**ED visit**: at least one	228,280 (40.7)
ED visit: High use (90th pct, i.e. > 3)	57,239 (10.2)
**Hospital admission**: at least one	325,513 (58.1)
Hospital admission: High use (90th pct, i.e. > = 2)	127,976 (22.8)
**Mortality (follow-up > =30 days)**	
Within first year	107,378 (19.2)
Within five years	219,315 (39.1)
Overall, during follow-up	281,268 (49.8)

^a^7503 with follow-up = 0; all deceased same day as cancer diagnosis (93% with at least another condition, 15% with 4 conditions and 30% with 5 or more conditions). ^b^ 40,761 with follow-up< 30 days (i.e. died within 30 days) excluded

### Cancer patients were likely to be complex, with high levels of MMB

**Fig. 1:**
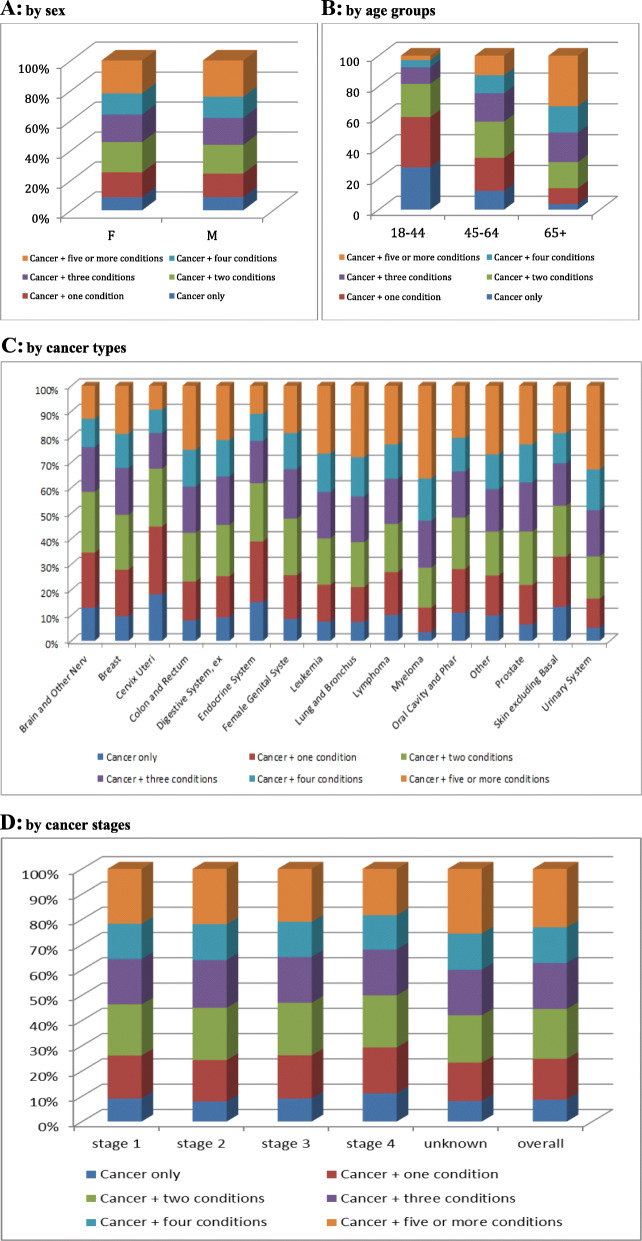
Multimorbidity level by sex, age, cancer site and stage

The majority of people with cancer (91.3%) had one or more co-occurring chronic conditions; nearly one quarter (23.2%) had five or more co-occurring conditions (Table 1). Complexity was similar between men and women, but more prevalent among those 65 or more years of age (Figs. 1a & b). MMB prevalence and levels varied across cancer types. For example, 9.4% of patients with cervix uteri cancer had five or more co-occurring conditions while 36.4% of people with myeloma had five or more co-occurring conditions (Fig. 1c). Moreover, we found that overall MMB was associated with stage at cancer diagnosis (Fig. 1d). For example, the average rate of no MMB (cancer only) was 8.7% in the population but 11.1% among people cancer-diagnosed at stage 4. Relatedly, people with cancer diagnosed at stage 4 had lower rates of extensive (i.e., 5 or more) MMB conditions (18.4%) compared to the overall population (23.2%). However, ‘unknown’ cancer stage at diagnosis accounted for a larger group among those with 5 or more MMB conditions than lower MMB levels. Also, those with higher levels of multimorbidity prior to or up to the time of cancer diagnosis (4, 5+) were more likely to be diagnosed at cancer stage 4.

Most people (84%) had MMB at the time of cancer diagnosis (before and up to 30 days after diagnosis). After cancer diagnosis, 45% of patients developed at least one additional condition during follow-up; rates of new chronic conditions after cancer diagnosis were similar (47%) among those with no MMB at the time of cancer diagnosis. Overall, 22% of the complex population received all MMB diagnoses within the first year of cancer, and 70% received all of their diagnoses within 5 years (results not shown).

### Health service utilization increased with MMB level, regardless of patient characteristics

Our design allowed us to follow individuals in the population for up to 15 years. During the follow-up period and, among those with at least 30 days of follow-up observation, 92% had at least one primary care visit, 41% had at least one ED visit, and 58% had at least one hospital admission within the first year (Table 1). Utilization rates during follow-up among those with at least one service visit was 6 primary care visits, 0.7 ED visits, and 0.5 hospitalizations per year, respectively. The proportion of patients with high use (90th percentile) increased with increasing MMB. For example, of those with five or more conditions, 22%, 14% and 28% were high users of PC, ED visits and hospitalizations, respectively  (data not shown).

**Fig. 2:**
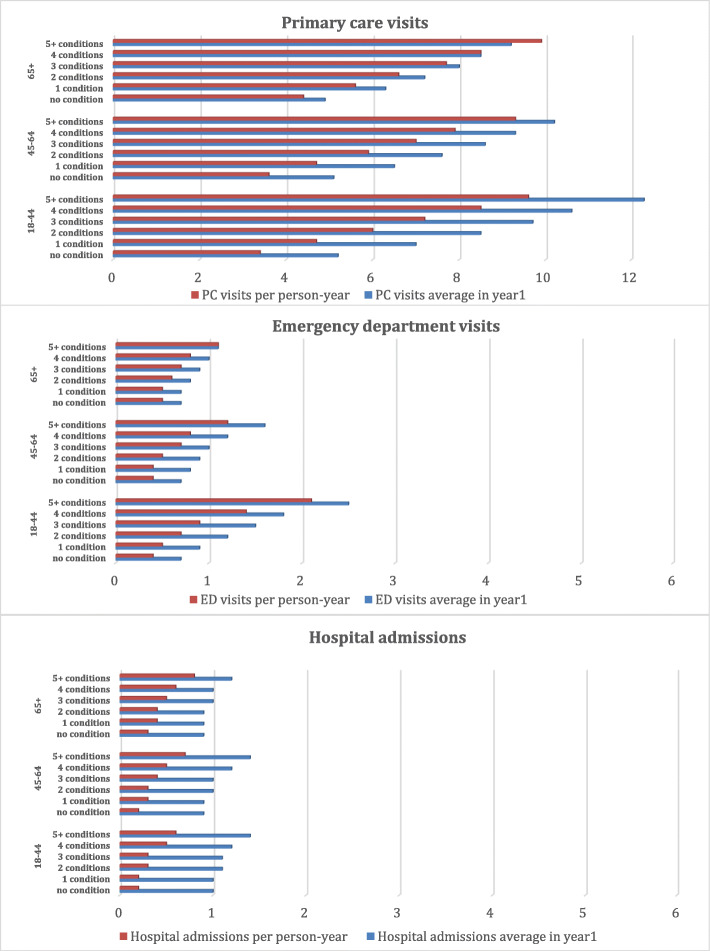
Health Service Utilization (HSU) by MMB level, prior to and following cancer diagnosis, by age, among those with at least 30 days of follow-up (*n* = 560,570)

HSU was the highest during the first year following cancer diagnosis. The number of PC visits, ED visits and hospitalizations increased with higher-levels of MMB in all age groups, in the first year and throughout the follow-up period (Fig. 2).

**Fig. 3:**
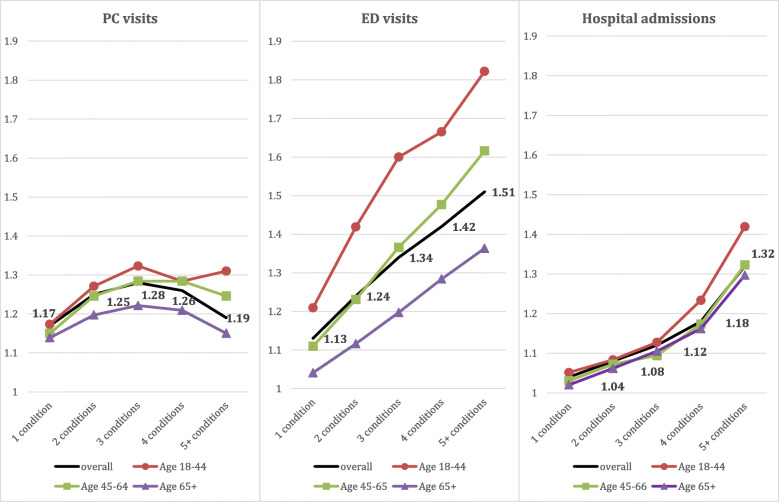
Adjusted impact of MMB on the number of health services encounters, overall and by age groups. Incidence rate ratio (IRR) values are displayed for the overall population and all analyses were adjusted for age, cancer type, stage, sex and number of visits one year before diagnosis

**Fig. 4:**
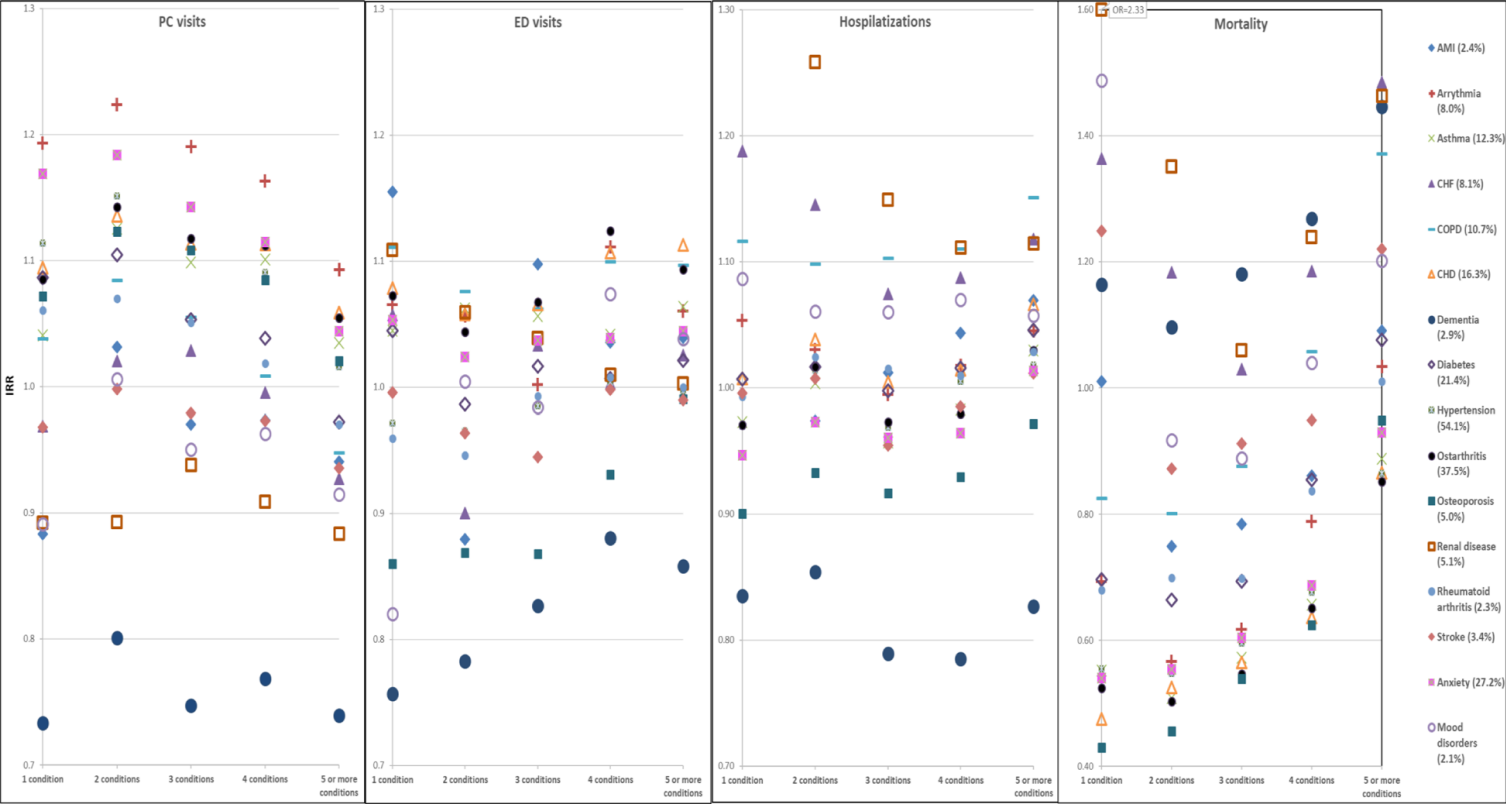
Incidence rate ratio (IRR) shows the impact of pre-existing individual conditions on health service use and mortality, by MMB levels. All analyses were adjusted for age, cancer type, stage, sex and number of visits one year before diagnosis. Prevalence of each condition in the study population is provided between bracket. For example, 5.1% of those who survived at least 30 days had been diagnosed with renal disease prior to cancer; 54.1% with hypertension, etc. As shown, impact of each condition greatly varies by MMB level and between conditions within the same level

The differences between MMB levels in terms of HSU were more substantial among younger adults, ages 18 to 44 years. In the adjusted models, increasing complexity was associated with higher rates of HSU, after controlling for age, cancer type, stage, sex and number of visits one year before diagnosis. While there was no clear gradient related to PC visits, those with five or more conditions beside cancer exhibited 51% and 32% more ED visits and hospital admissions respectively than those with cancer only (Fig. 3). Individual co-occurring conditions had varying impacts on HSU; however, the effects of individual conditions appeared less in the presence of higher levels of MMB (Fig. 4).

Overall, there was a clear gradient in the impact of cancer stage on all HSU measures, except PC visits (Additional file 1).

### Cancer survival and mortality was associated with cancer type and age, and MMB had an incremental, independent negative impact

**Fig. 5:**
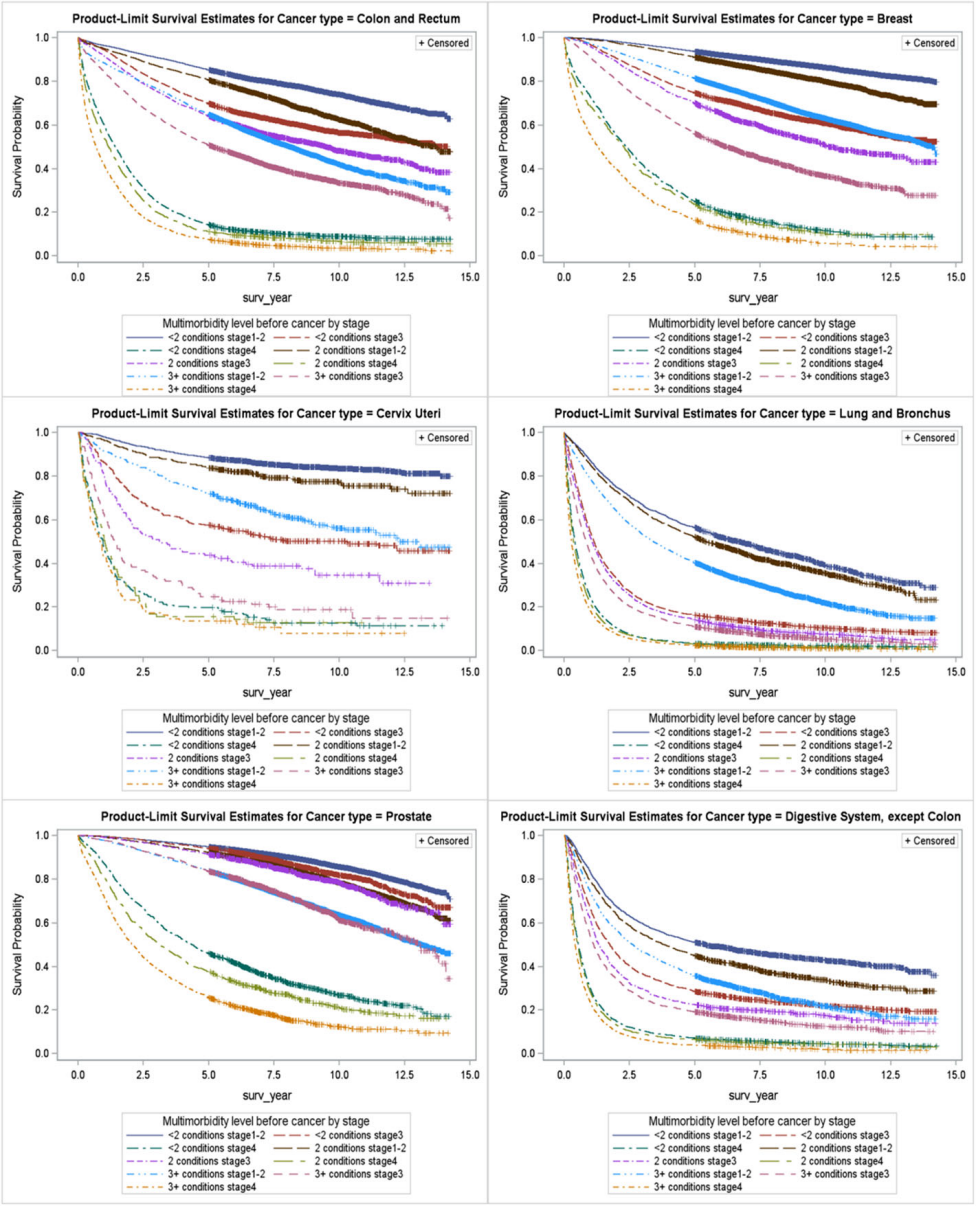
Kaplan-Meier survival curves by multimorbidity and stage at diagnosis for selected cancer types

The overall five-year survival rate among the study population followed for at least 30 days was 61%, ranging from 18 to 95% by cancer type. The incremental impact of MMB, however, was consistent and negative for cancer types with similar prognoses. The impact was apparent at any stage, but differences between MMB levels were more pronounced at earlier stages of diagnosis. An exception was the limited impact of MMB on survival among people with lethal cancer types, where diagnosis was often at later stage (Fig. 5).

About half of the population died during follow-up and the mortality rate within one year was 19.2% (Table 1). Early death occurred mostly among those with lung/bronchus, digestive system (except colon) and brain and other nervous system cancers (data not shown). The risk of dying within the first year among individuals with between one and three co-occurring conditions was lower or similar to those with no condition prior to cancer diagnosis, whereas those with five or more conditions were 45% more likely to die, regardless of age, sex, cancer type or stage (Additional file 1). Mortality was higher for stage 2 (IRR = 2.3) and stage 4 (IRR = 23.3) compared to stage 1.

The impact of individual chronic conditions was largely different within and between levels of MMB. For example, the risk of death varied from 0.43 (osteoporosis) to 2.33 (renal disease) among those with one condition prior to cancer, and from 0.85 (hypertension) to 1.49 (CHF) among those with five or more conditions prior to cancer (Fig. 4). Surprisingly, COPD decreased the risk of death by 17% when present alone before cancer, while increasing the risk of death by 37% when present with at least four other conditions.

Detailed results of the adjusted impact of MMB on HSU and mortality overall and by age group are in Additional file 1 - Appendices 1 and 2.

## Discussion

This population-level study is among the first to examine the co-occurrence of cancer of all types and 16 other chronic conditions managed outside of the cancer care system. While research on MMB continues to grow, there are only few studies assessing the number of co-occurring conditions among people with cancer, and those studies have typically included only a few specific cancer sites [7, 8, 11]. By describing the extent of MMB within cancer of all types, we aim to inform Canadian and global efforts to improve quality, efficiency, and patient outcomes and experiences in complex (i.e., multimorbid) cancer care.

Overall, the prevalence of MMB in cancer patients is among the highest [4, 41]. Similar to previous studies [7, 11], we found that cancer MMB was extremely common (91%) in our population of patients diagnosed with cancer between 2003 and 2013. MMB was also present across all stages of cancer, with some indication that MMB prior to cancer was related to diagnosis at later stages, but the overall proportion of extensive MMB was lower at later cancer stage, likely due to survival bias or lack of focus on other conditions following cancer diagnosis. Our data also showed that rates of cancer MMB remained stable over time, indicating the ongoing need to address MMB effectively.

While patients are likely to exhibit multiple conditions before cancer, MMB continues to increase substantially after a cancer diagnosis. In our study, we found that nearly half of all people with cancer were diagnosed with an additional chronic condition during follow-up. This is consistent with data from Leach and colleagues [7] in which cancer patients reported the emergence of approximately 1.9 new conditions after diagnosis of one of five common cancer types.

Our data is also consistent with previous studies showing no sex difference in MMB rates but increased rates and levels of MMB among older age groups [9, 11]. Notable from our study, however, are findings that cancer MMB was observed across all age groups, including high rates of cancer MMB  with two or more conditions in young adults (ages 18–44 years). This suggests that MMB is not only an issue for older adults but a growing issue for adults of all ages [4, 41–43].

Cancer sites with the highest prevalence of MMB were myeloma, prostate, urinary system, lung/bronchus and Leukemia. For example, those with four or more conditions beside cancer represented a substantial proportion for all sites of cancer from 19% (cervical cancer) to 53% (myeloma). Similar to our study, Fowler et al. [11] found that MMB was higher among people with lung cancer compared to the other four cancers included in their study, with 67% of lung cancer patients having one or more comorbidities. Different from Fowler and colleagues, however, is that our overall rates of MMB are higher. An important difference between our methods is that Fowler et al. [11] considered four possible co-occurring conditions instead of the 16 included here. Other contextual factors, such as public health screening initiatives, may also be important for between-population and between-cancer rates of MMB. For example, in Ontario, Canada, cervical cancer was among the sites with the lowest prevalence of MMB in our study (66% before cancer and 82% overall) but it is also the target of a province-wide screening initiative for women as young as age 21 [44] who are less likely than older adults to have MMB.

We also analyzed the impact of MMB on multiple aspects of the HSU. While adequate access to primary care may contribute to better care management and patient outcomes, high use of ED and hospital services, and ultimately death, reflect poor patient health which may be aggravated by the presence of multiple conditions. Not surprisingly, both mortality and HSU were positively associated with increasing MMB levels in our study, regardless of patients’ age, sex, cancer type or cancer stage. This is in line with previous research showing that higher MMB was associated with a higher risk of death or lower survival among patients with specific cancer types [6, 12, 45]. Legler et al. (2011) also found a positive association between high patient Charlson Comorbidity Index and increased admission to ED, hospital, and ICU [46]. Unlike prior work, a unique contribution of this paper is that we examined the relationship between MMB level and HSU. In those analyses, we observed that increasing MMB level had a greater negative impact on HSU among younger people: the difference between cancer only and the highest level of MMB was on average 1.8 ED visits and 7.1 PC visits in young adults compared to 0.4 and 4.3 among people 65 years and older. The trend was opposite for mortality: increasing MMB was not as strongly linked to risk of death among younger adults. More research examining potential contributing factors, such as early screening and identification through regular health service use, for example, is needed to better understand cancer MMB in younger adults.

Lastly, our data showed that individual conditions had varied impacts on patients’ health outcomes, depending on the level of MMB. Conditions frequently identified as co-occurring with cancer in previous studies include hypertension, COPD, diabetes, CVD, and CHF [11, 46]. These conditions were also among the most frequent in our study population. The most common co-occurring conditions (hypertension, arthritis, anxiety, diabetes) often had the greatest impact on outcomes; however, other less prevalent conditions are also worth considering. Overall, the relationship between cancer, mental health (including substance use disorder) diagnoses, and HSU is in need of further study. Our data showed increased PC encounters among people with co-occurring anxiety disorder, which was also associated with fewer hospitalizations and a lower risk of death. While it well known that psychiatric conditions are a significant driver of HSU (e.g., [47]), conditions like anxiety that increase preventive contacts with the healthcare system may represent a protective form of MMB. Other conditions such as dementia or psychosis that limit patient capacity for self-care, in comparison, may increase higher-acuity service use, such as ED visits and hospitalizations, signaling the need for more intensive preventive care and/or illness self-management supports. Overall, these findings suggest that while MMB is important to understand overall, unique combinations of co-occurring conditions are likely to have differential effects on HSU and patient outcomes, and thus require further study.

### Strengths and limitations

This is the first population-based cohort study to examine the burden of MMB and its impact in the Ontario population of people with all types of cancer. It presents many strengths, including the size of the study population, extended follow-up of between five and 15 years, and the use of administrative data including multiple chronic conditions, including indicators of mental health. Because Ontario has universal health coverage, Ontario’s health administrative data provide robust, population-based estimations of cancer MMB [48] not available from self-report or other sources. In fact, with comprehensive health administrative data, estimates of MMB are more likely to be reliable and complete than patient self-report [49]. In this study, we operationalized MMB through 16 high-burden chronic conditions, both prior to cancer and for five or more years following cancer diagnosis. Most of these chronic conditions were operationalized using validated algorithms; however, some conditions may not be adequately represented [50, 51]. Namely, studies have found mixed results related to the under- or over-diagnosis of co-occurring conditions in cancer patients [52, 53]. Though the number of conditions is adequate to assess overall MMB [54], another limitation is that other potentially relevant chronic conditions were not included which could have unique effects as individual diseases and/or as part of the effects of MMB level. Another limitation of this report is that the severity of non-cancer conditions is not considered in our assessment.

In addition to our main objective of describing MMB in cancer patients, we also aimed to advance the understanding of MMB on HSU and mortality. To do this, our analysis included only basic patient demographics and an indicator of cancer severity, which is sufficient to describe baseline impacts of MMB and develop hypotheses and rationale for further research. That said, we acknowledge that many other potentially confounding variables, such as socioeconomic status, could have potentially impacted our results and as such may be important in future work. There may also be some residual confounding regarding cancer stage because of the limitations in the staging data and the large proportion of missing information (presented as unknown); however, we believe that this approach is adequate to support the exploratory nature of this work. We also did not examine treatment approaches and quality of care that can play a crucial role in cancer outcomes.

## Conclusions & Implications

People with cancer are very likely to experience MMB over all and at very high levels, regardless of age and cancer type – including young adults whose risk for MMB may have been previously overlooked. The findings in this report can inform health system advances towards person-centered care as it describes the overall nature MMB for the whole range of cancer types. At the same time, our findings also show that there is wide variation in the impact of individual conditions within and between MMB levels, suggesting that it is crucial to assess the role of each condition within a MMB lens. This could, for example, include routine assessment of chronic conditions that commonly co-occur with cancer and coordinated care pathways that reduce treatment burden and increase access to targeted supports (e.g., mental health; illness self-management) for patients likely to experience the need. More generally, however, our findings show that at higher levels of MMB, individual conditions have less of an impact on outcomes that MMB itself, again highlighting the need to improve how care for people with MMB is envisioned and operationalized. In the next paper, we describe the relationship between specific, observed clusters of cancer and chronic diseases and their relationship to HSU and mortality risk to help guide next steps in advancing MMB care.

## Supplementary Information

**Additional file 1: Appendix 1.** Adjusted impact of MMB on HSU and mortality among patients with cancer and at least 30 days of follow-up (*n* = 560,570). **Appendix 2**. Adjusted impact of MMB on HSU and mortality among patients with cancer and at least 30 days of follow-up (*n* = 560,570), stratified by age groups.

## Data Availability

The data that support the findings of this study are available from ICES but restrictions apply to the availability of these data, which were used under the service agreement ICES DAS # 2020–727, and so are not publicly available. Access may only be granted under specific criteria and confidentiality conditions; please see www.ices.on.ca/DAS. The contact person is Refik Saskin, Staff Scientist, Data & Analytics Services ICES Central, (1) 416–480-4055.

## Abbreviations

CHF: Congestive heart failure
COPD: Chronic obstructive pulmonary disease
CVD: Cardiovascular disease
DAD: Discharge Abstract Database
ED: Emergency Department
HSU: Health services utilization
ICES: Institute for Clinical and Evaluative Sciences
ICD-O-3: International Classification of Diseases for Oncology, 3rd version
MMB: multimorbidity
NACRS: National Ambulatory Care Reporting System
ODB: Ontario Drug Benefits
OHIP: Ontario Health Insurance Plan
PC: Primary Care
RPDB: Registered Persons Database
SEER: Surveillance, Epidemiology, and End Results

## Declarations

### Methods

All methods were carried out in accordance with relevant guidelines and regulations, including data acquisition, cleaning, analyses and reporting of findings.
