# Supplementary material for: Prevalence of multimorbidity in adults with cancer, and associated health service utilization in Ontario, Canada: a population-based retrospective cohort study
Source: BMC Cancer. 2021 Apr 14;21:406. doi: 10.1186/s12885-021-08102-1 (PMC8048167; doi:10.1186/s12885-021-08102-1)
Supplement: Supplementary file 1 — Additional file 1: Appendix 1. Adjusted impact of MMB on HSU and mortality among patients with cancer and at least 30 days of follow-up (n = 560,570). Appendix 2. Adjusted impact of MMB on HSU and mortality among patients with cancer and at least 30 days of follow-up (n = 560,570), stratified by age groups. [file 12885_2021_8102_MOESM1_ESM.docx]

**Appendix 1. Adjusted impact of MMB on HSU and mortality among patients with cancer and at least 30 days of follow-up (n= 560,570)**

|  | **PC visits** | **ED visits** | **Hospital admissions** | **Mortality** |
| --- | --- | --- | --- | --- |
|  | **IRR** | **IRR** | **IRR** | **OR** |
| **Multimorbidity before cancer (ref= no condition)** |  |  |  |  |
| 1 condition | 1.17 | 1.13 | 1.04 | 0.90 |
| 2 conditions | 1.25 | 1.24 | 1.08 | 0.92 |
| 3 conditions^1^ | 1.28 | 1.34 | 1.12 | 0.99 |
| 4 conditions | 1.26 | 1.42 | 1.18 | 1.11 |
| 5+ conditions | 1.19 | 1.51 | 1.32 | 1.45 |
| **Cancer type (ref= Other type)** |  |  |  |  |
| Brain and Other Nervous System^2^ | 1.00 | 1.17 | 1.23 | 3.57 |
| Breast | 1.32 | 1.07 | 0.71 | 0.23 |
| Cervix Uteri^3^ | 1.03 | 0.96 | 1.09 | 1.15 |
| Colon and Rectum^3^ | 1.20 | 1.01 | 1.17 | 0.53 |
| Digestive System, except Colon and Rectum | 0.98 | 1.11 | 1.25 | 2.92 |
| Endocrine System | 1.13 | 0.59 | 0.90 | 0.11 |
| Female Genital System, except Cervix | 1.12 | 0.96 | 1.14 | 0.70 |
| Leukemia^2,3^ | 1.01 | 1.00 | 1.21 | 0.78 |
| Lung and Bronchus^3^ | 0.97 | 1.01 | 1.07 | 2.88 |
| Lymphoma^3^ | 1.05 | 1.01 | 0.92 | 0.60 |
| Myeloma | 1.08 | 1.20 | 1.28 | 0.68 |
| Oral Cavity and Pharynx | 1.14 | 0.94 | 1.13 | 0.48 |
| Prostate | 1.18 | 0.71 | 0.54 | 0.12 |
| Skin excluding Basal and Squamous | 1.11 | 0.78 | 0.37 | 0.26 |
| Urinary System^3^ | 1.14 | 0.99 | 1.05 | 0.47 |
| **Cancer Stage (ref= 1)** |  |  |  |  |
| 2 | 1.02 | 1.22 | 1.27 | 2.29 |
| 3 | 1.08 | 1.57 | 1.63 | 5.11 |
| 4 | 0.86 | 1.53 | 1.67 | 23.31 |
| unknown | 0.93 | 1.10 | 1.16 | 6.92 |
| **Age (in years)** | 0.99 | 0.99 | 0.99 | 1.05 |
| **Male sex** | 1.03 | 1.10 | 1.10 | 1.12 |
| **Number of visits one year before diagnosis** | 1.06 | 1.26 | 1.05 |  |
| **Dispersion** | 0.43 | 1.58 | 0.30 |  |
| **Deviance** | 1.14 | 0.86 | 1.02 |  |
| **Pearson Chi-Square** | 1.05 | 1.34 | 1.07 |  |
| **c-statistic** |  |  |  | 0.87 |
| **Max-rescaled R-Square** |  |  |  | 0.42 |

All associations are significant with p <0.001, except in the following cases:

^1^ non-significant for Mortality; ^2^ non-significant for PC visits; ^3^ non-significant for ED visits

**Appendix 2: Adjusted**^1^ **impact of MMB on HSU and mortality among patients with cancer and at least 30 days of follow-up (n= 560,570), stratified by age groups**

|  | PC visits | ED visits | Hospital admissions | Mortality |
| --- | --- | --- | --- | --- |
| **Age 18-44** | IRR | IRR | IRR | OR |
| Multimorbidity before cancer (ref= no condition) |  |  |  |  |
| 1 condition | 1.17 | 1.21 | 1.05 | 0.99 |
| 2 conditions | 1.27 | 1.42 | 1.08 | 0.99 |
| 3 conditions | 1.32 | 1.60 | 1.13 | 1.08 |
| 4 conditions | 1.28 | 1.67 | 1.23 | 1.22 |
| 5+ conditions | 1.31 | 1.82 | 1.42 | 1.55 |
| **Age 45-64** | PC visits | ED visits | Hospital admissions | Mortality |
|  | IRR | IRR | IRR | OR |
| Multimorbidity before cancer (ref= no condition) |  |  |  |  |
| 1 condition | 1.15 | 1.11 | 1.03 | 1.00 |
| 2 conditions | 1.25 | 1.23 | 1.07 | 1.21 |
| 3 conditions | 1.28 | 1.37 | 1.09 | 1.17 |
| 4 conditions | 1.28 | 1.48 | 1.17 | 1.84 |
| 5+ conditions | 1.25 | 1.62 | 1.32 | 2.27 |
| **Age 65+** | PC visits | ED visits | Hospital admissions | Mortality |
|  | IRR | IRR | IRR | OR |
| Multimorbidity before cancer (ref= no condition) |  |  |  |  |
| 1 condition | 1.14 | 1.04 | 1.02 | 0.86 |
| 2 conditions | 1.20 | 1.12 | 1.06 | 0.90 |
| 3 conditions | 1.22 | 1.20 | 1.11 | 0.96 |
| 4 conditions | 1.21 | 1.28 | 1.16 | 1.06 |
| 5+ conditions | 1.15 | 1.36 | 1.30 | 1.37 |

^1^All analyses were adjusted for age, cancer type, stage, sex and Number of visits one year before diagnosis
